# Hemokinin-1(4-11)-Induced Analgesia Selectively Up-Regulates δ-Opioid Receptor Expression in Mice

**DOI:** 10.1371/journal.pone.0090446

**Published:** 2014-02-28

**Authors:** Cai-Yun Fu, Rui-Long Xia, Teng-Fei Zhang, Yan Lu, Shi-Fu Zhang, Zhi-Qiang Yu, Tao Jin, Xiao-Zhou Mou

**Affiliations:** 1 Lab of Proteomics and Molecular Enzymology, College of Life Sciences, Zhejiang Sci-Tech University, Hangzhou, China; 2 Zhejiang Provincial People’s Hospital, Hangzhou, China; 3 Institute for Cell-Based Drug Development of Zhejiang Province, Hangzhou, China; 4 Center for BioEnergetics, The Biodesign Institute, and Department of Chemistry and Biochemistry, Arizona State University, Tempe, Arizona, United States of America; University of Arizona, United States of America

## Abstract

Our previous studies have shown that an active fragment of human tachykinins (hHK-1(4-11)) produced an opioid-independent analgesia after intracerebroventricular (i.c.v.) injection in mice, which has been markedly enhanced by a δ OR antagonist, naltrindole hydrochloride (NTI). In this study, we have further characterized the *in vivo* analgesia after i.c.v. injection of hHK-1(4-11) in mouse model. Our qRT-PCR results showed that the mRNA levels of several ligands and receptors (e.g. PPT-A, PPT-C, KOR, PDYN and PENK) have not changed significantly. Furthermore, neither transcription nor expression of NK_1_ receptor, MOR and POMC have changed noticeably. In contrast, both mRNA and protein levels of DOR have been up-regulated significantly, indicating that the enhanced expression of δ opioid receptor negatively modulates the analgesia induced by i.c.v. injection of hHK-1(4-11). Additionally, the combinatorial data from our previous and present experiments strongly suggest that the discriminable distribution sites in the central nervous system between hHK-1(4-11) and r/mHK-1 may be attributed to their discriminable analgesic effects. Altogether, our findings will not only contribute to the understanding of the complicated mechanisms regarding the nociceptive modulation of hemokinin-1 as well as its active fragments at supraspinal level, but may also lead to novel pharmacological interventions.

## Introduction

Belonging to a family of closely related peptides, tachykinins share a common C-terminal sequence (FXGLM-NH_2_). Tachykinins are widely distributed within mammalian peripheral and central nervous systems [1]. The best known members of the tachykinin family are substance P (SP), neurokinin A (NKA) and neurokinin B (NKB), that are derived from two distinct genes: *Ppta* encodes for SP, NKA, and two additional extended forms of NKA (NPK and NPγ) [2], *Pptb* encodes for NKB [3]. Previously, a third preprotachykinin gene, *Pptc* encoding hemokinin-1 (HK-1), has been discovered [4], [5]. First identified in hematopoietic cells from mice, HK-1 has been demonstrated to have an important role in the maturation from pre-B to pro-B cells [5]. Later, HK-1 has also been found in rats and humans [4]. The rat *Pptc* polypeptide is highly homologous to mouse *Pptc* and contains the same processing sites to generate the predicted HK-1 (r/mHK-1, RSRTRQFYGLM-NH_2_). The human *Pptc* polypeptide has been predicted to contain two potential monobasic cleavage sites rather than a single dibasic site, so as to alternatively produce full-length HK-1 (as hHK-1, TGKASQFFGLM-NH_2_) or a truncated version (as hHK-1(4-11), ASQFFGLM-NH_2_) [4].

Accumulating evidence has suggested that human and mouse HK-1 peptides were nearly identical to SP in their overall activity profile on three NK receptors with the highest affinity for NK_1_ receptor [4], [6], [7]. However, unlike r/mHK-1 and SP, which interact with NK_1_ receptor with similar binding affinities, hHK-1 or hHK-1(4-11) showed somewhat attenuated affinities (14- and 70-fold, respectively) [4]. To date, a wide range of human/mouse HK-1 activities have been investigated, including immunological system [5], [8], cardiovascular system [9]–[14], and pain sensation system [15]–[20]. Particularly in modulation of pain transmission, our previous work has shown that r/mKH-1 [15], hHK-1 and hHK-1(4-11) [19] all could produce a dose- and time-dependent antinociceptive effect after intracerebroventricular (i.c.v.) administration, and the antinociceptive effect induced by r/mKH-1 and hHK-1 was significantly blocked by the antagonists of NK_1_ receptor and μ opioid receptor, respectively [19]. Intriguingly, the antinociceptive effect induced by hHK-1(4-11) was not affected by these two kinds of antagonists. On the contrary, blocking of δ opioid receptor significantly enhanced the analgesia of hHK-1(4-11), indicating that δ opioid receptor could be a negative modulatory factor in the analgesic effect of hHK-1(4-11) [19]. Our previous results thus showed obvious differences between the analgesic effects induced by HK-1 and hHK-1(4-11). In this study, we further investigated the distribution sites and the molecular mechanisms after i.c.v. administration of hHK-1(4-11) in more details, in comparison with our previous publication [21].

## Materials and Methods

### Animals

ICR mice (20±1.0 g) were supplied regardless of gender by the animal center of Hangzhou Normal University (Hangzhou, China). All animal experimental protocols were approved by the Animal Care and Use Committee of the Zhejiang Sci-Tech University, and were in compliance with the European Communities Council Directive of 24 November 1986 (86/609/EEC). All animals were kept at 23°C∼25°C with a 12-hour light/dark cycle and allowed standard chow and water until the time of the study. Every effort was made to minimize the number as well as suffering of sacrificed animals in the experiments.

### Peptides

Fluo-hHK-1(4-11) and hHK-1(4-11) peptides were synthesized by Chinese Peptide Company (Hangzhou, China) using the solid-phase peptide method and purified by high-performance liquid chromatography (HPLC) with a purity of more than 98%. FAM was carboxyfluorescein, and the standard coupling method was used to couple 5-carboxyfluorescein to the amino group of the hHK-1(4-11) peptide [22]. The peptide hHK-1(4-11) was dissolved in normal saline at a working concentration of 1.5 mM. Fluorescein-labeled (FAM) hHK-1(4-11) was solubilized in 50% dimethyl sulfoxide (DMSO) at the concentration of 15 mM for stock solution. Then, it was diluted in normal saline at the working concentration of 1.5 mM with 5% DMSO before the experiments.

### Intracerebroventricular Injection and Tissue Preparation

Intracerebroventricular (i.c.v.) injection was performed following the method described by Haley and McCormick in conscious mice [23]. The injection site was similar to our recent report [21]. The final dose of hHK-1(4-11) or fluo- hHK-1(4-11) was 6 nmol per mouse administered in a volume of 4 µl according to our previous report [19].

Our previous study showed that the analgesia after i.c.v. administration of hHK-1(4-11) was maintained within 20 min, so the time points of 5, 10 and 20 min were selected for this research. After injection of FAM-hHK-1(4-11) or hHK-1(4-11) (6 nmol per mouse), the mice were sacrificed by decapitation at each time point, and the proper injection site was verified by microscopic measurement. In the control group, 4 µl of normal saline were injected into the sites of i.c.v. in mice. Then, the brains administrated with hHK-1(4-11) or normal saline were quickly removed and stored at −80°C until further analysis. The brains administrated with FAM-hHK-1(4-11) were quickly removed and fixed in 4% paraformaldehyde (Sigma) in 0.1 M sodium phosphate buffer at pH 7.4 for 24 h, and cryoprotected with 30% sucrose before OCT (JUNG Tissue freezing medium, LEICA,Germany) embedding and freezing.

### Coronal Sections and Light Microscopy

The procedures of coronal sections and light microscopy for each brain were consistent with the procedures in our recently published work [21]. Briefly, each of the embedded brains using OCT was frozen at −20°C in a LEICA CM1900 cryostat and sectioned coronally into 10 µm slices. Then, the freshly cut sections were placed individually on pre-cooled (−20°C) microscopy slides using cool tweezers. Sections were stored at 4°C, but for no longer than 12 h before observation. Samples were observed and photographed in an inverted phase contrast and fluorescence microscope (NIKON TE2000-U). Anatomical structures were identified according to an adult mouse brain atlas [24].

### Reverse Transcription and Real-time Polymerase Chain Reaction

Total RNA from each frozen brain was extracted using TRIzol Reagent (Invitrogen, Carlsbad, CA) according to the manufacturer’s instructions. Briefly, approximately 100 mg of frozen brain tissue was homogenized in 1 ml TRIzol reagent using a mortar and pestle until they became red-violet. In all experiments, 5 µg of total RNA was reverse-transcribed into cDNA using oligo dT (12–18 mer, Gibco BRL) in a final volume of 50 µl. The primer sequences were as follows, which are the same as in our published work [21]:

*NK_1_ receptor* (F): 5′-TGGACTCTGATCTCTTCCCCAACA-3′

*NK_1_ receptor* (R): 5′-GGACCCAGATGACAAAGATGACCACTT-3′

*PPT-C* (F): 5′-CGGGCCATCAGTGTGCACTA-3′

*PPT-C* (R): 5′-GGAATCCCCGTCCCCAGCAT-3′

*PPT-A* (F): 5′-GAAATCGATGCCAACGATGATC-3′

*PPT-A* (R): 5′-AGGCTTGGGTCTTCGGGCGATTCT-3′

*MOR* (F): 5′-ATCCTCTCTTCTGCCATTGGT-3′

*MOR* (R): 5′-TGAAGGCGAAGATGAAGACA-3′

*POMC* (F): 5′-AGATTCAAGAGGGAGCTGGA-3′

*POMC* (R): 5′-CTTCTCGGAGGTCATGAAGC-3′

*KOR* (F): 5′-CCGATACACGAAGATGAAGAC-3′

*KOR* (R): 5′-GTGCCTCCAAGGACTATCGC-3′

*PDYN* (F): 5′-CGGAACTCCTCTTGGGGTAT-3′

*PDYN* (R): 5′-TTTGGCAACGGAAAAGAATC-3′

*DOR* (F): 5′-AAGTACTTGGCGCTCTGGAA-3′

*DOR* (R): 5′-GCTCGTCATGTTTGGCATC-3′

*PENK* (F): 5′-AACAGGATGAGAGCCACTTGC-3′

*PENK* (R): 5′-CTTCATCGGAGGGCAGAGACT-3′

*GAPDH* (F): 5′-AGGAGCGAGACCCCACTAACAT-3′

*GAPDH* (R): 5′-GTGATGGCATGGACTGTGGT-3′

Relative quantitative real-time PCR was used to assess the mRNA levels of these above genes (ABI 7300 real-time PCR detection system), in which GAPDH was used as an internal control. Negative controls consisted of samples without a DNA template. After each real-time quantitative PCR, a dissociation curve analysis was conducted. The normalized expression level of the target gene was calculated by the 2^−ΔΔCt^ method [25], where ΔΔCt = (Ct,_Target_ −Ct,_GAPDH_)_Time x_−(Ct,_Target_−Ct,_GAPDH_)_Time 0_. Time x is indicated any time point (5, 10 and 20 min) and time 0 represents the expression of the target gene normalized to GAPDH of control (normal saline group). All reactions were run in triplicate and independently repeated at least three times. Statistical results were presented as mean ±standard error of mean (SEM).

### Western Blotting

Proteins were extracted from the mouse brain in accordance with our published work [21]. Protein concentrations were estimated using Coomassie Blue protein assay reagent (Pierce Chemical Co.), with concentration-known solutions of bovine serum albumin (BSA, Sigma) as a standard.

Protein samples were subjected to denaturing SDS polyacrylamide gel electrophoresis (SDS-PAGE) and subsequently electrotransferred onto PVDF membranes. The membrane was blocked at room temperature in TBST with 5% nonfat dry milk for 1 h, subsequently, incubated separately by primary antibodies of β-actin (1∶10000, Bioworld), POMC (1∶10000, ABGENT), MOR (1∶10000, Epitomics), NK_1_ receptor (1∶500, Proteintech) and DOR (1∶500, Bioworld) at 4°C overnight. The membranes were treated with HRP-anti-rabbit IgG (1/5000, Bioworld) for β-actin, DOR, MOR and TACR1 primary antibodies or HRP-anti-goat IgG (1/5000, Bioworld) for POMC primary antibodies. Target proteins were visualized by secondary antibodies by Chemiluminescent and Florescent imaging system (Sage Creation). The Western blotting was analyzed by quantifying pixel density with reference to the control (as 100%) (Quantity One software, Bio-Rad).

### Statistical Analysis

All analyses were independently done in no less than three replicates. Data are presented as mean±SEM. All statistical analyses were performed using the SPSS statistical package, version 16.0 (SPSS Inc., Chicago, IL, USA) for Windows. A value of *p*<0.05 was considered to be statistically significant.

## Results

### The Distribution Sites of hHK-1(4-11) during the Modulation of Pain after i.c.v. Administration in Conscious Mice

Our previous work indicated that i.c.v. administration of hHK-1(4-11) at the dosages of 0.3, 1, 3 and 6 nmol per mouse produced dose-dependent analgesia in tail-flick latencies, while NTI (a δ OR antagonist) markedly enhanced the analgesia effect induced by h HK-1-(4-11) [19]. In the present study, the dosage of 6 nmol per mouse was selected for further investigation. Additionally, the analgesic intensity of hHK-1(4-11) (6 nmol per mouse) verified in this study was consistent with our previous finding by the tail-flick test (data not shown).

**Figure 1:**
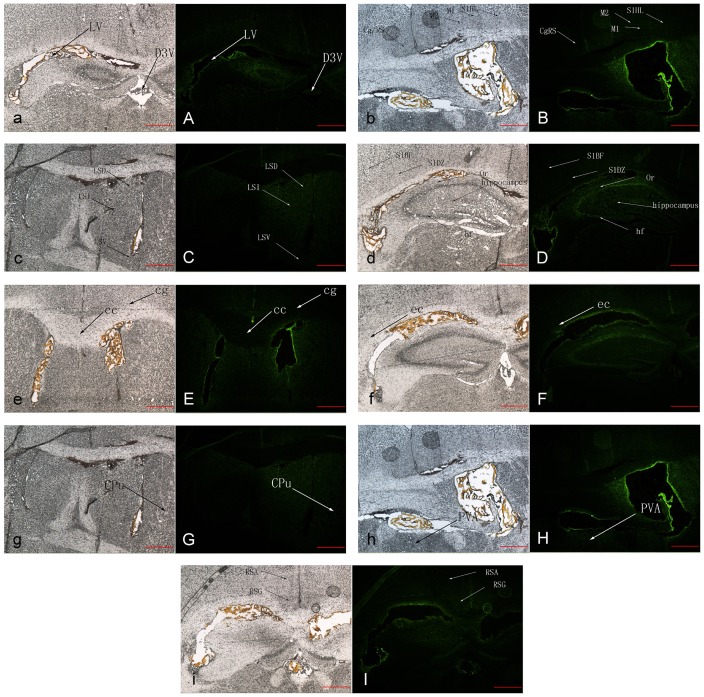
The distribution sites after i.c.v. administration of hHK-1(4-11) in mice. Representative photographs observed with the phase contrast microscope (NIKON TE2000-U, a–i) from at least three independent experiments. The identical coronal sections observed with the fluorescence microscope (NIKON TE2000-U, A–I) corresponding to the phase-contrast photographs of a–i. Scale bar = 50 µm. FAM-hHK-1(4-11): green. LV (lateral ventricle), D3V (dorsal 3rd ventricle),Cg/RS (cingulate/retrosplenial cortex), M1 (primary motor cortex), M2 (secondary motor cortex), S1HL(S1 cx, hindlimb region (primary somatosensory cortex, hindlimb region)), LSD (lateral septal nucleus, dorsal part), LSI (lateral septal nucleus, intermediate part), LSV (lateral septal nucleus, ventral part), S1BF (primary somatosensory cortex, barrel field), S1DZ (primary somatosensory cortex, dysgranular region), Or (oriens layer of the hippocampus), hf (hippocampal fissure), cc (corpus callosum), cg (cingulum), ec (external capsule), CPu (caudate putamen (striatum)), PVA (paraventricular thalamic nucleus, anteriorpart), RSA (retrosplenial agranular cortex), RSG (retrosplenial granular cortex).

As described in our previous work [21], the fluo-hHK-1(4-11) peptides were green fluorescent under blue light using the fluorescence microscope. At the time point of 10 min after i.c.v. administration, the fluo-hHK-1(4-11) peptides were distributed mainly at the cerebroventricular walls, as well as several juxta-ventricular structures (Figure 1). However, the main difference of the distribution sites between fluo-hHK-1(4-11) and fluo-r/mHK-1 was that there was no blue light in the structure of periaqueductal central gray (PAG) and E/OV (ependymal and subendymallayer/olfaetory) in the fluo-hHK-1(4-11) treated group.

### Characterization of the mRNAs Encoding NK_1_ Receptors and Different Preprotachykinins

**Figure 2:**
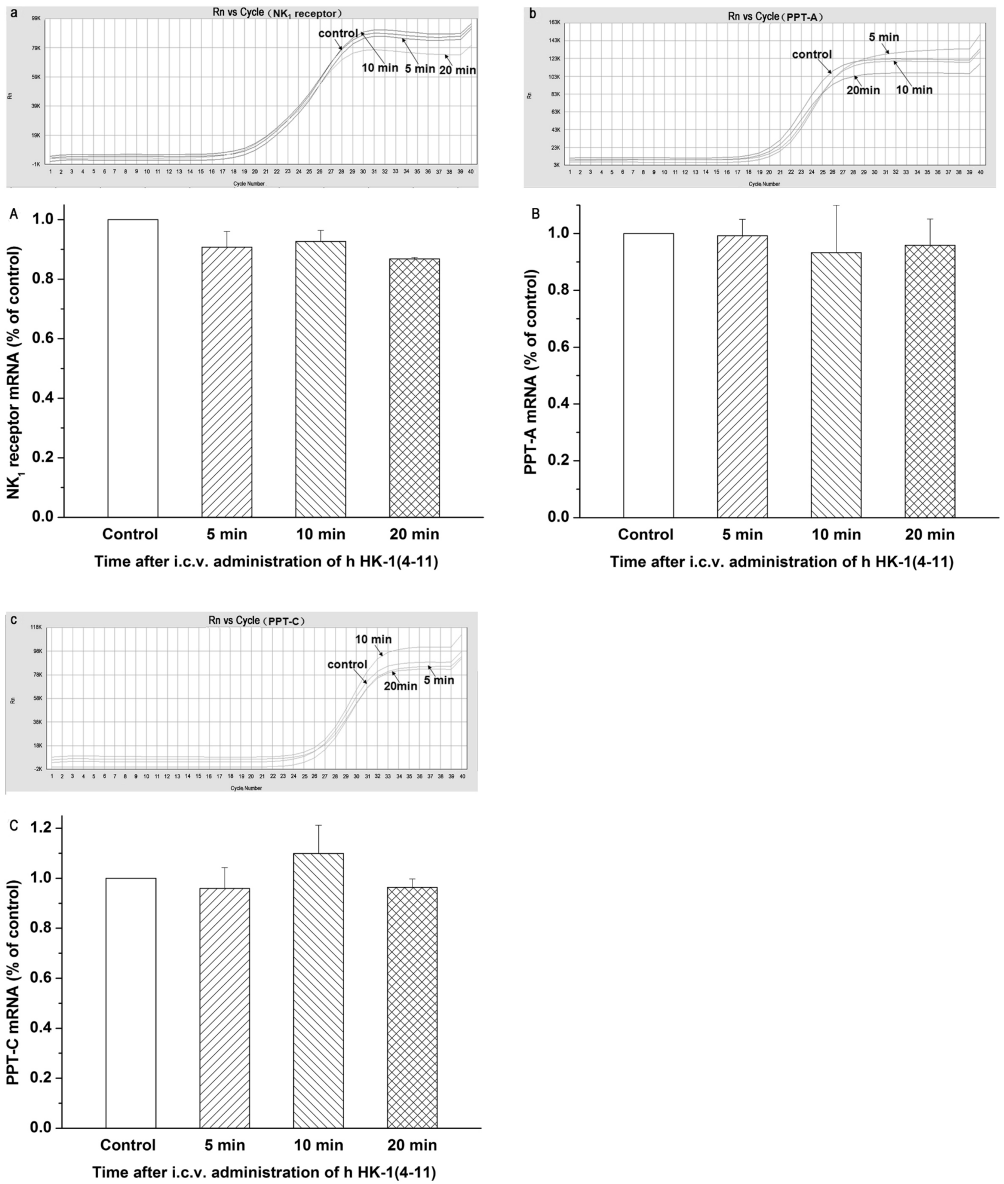
Quantitative determination of the mRNA expression levels of the NK_1_ receptor and the different preprotachykinins after i.c.v. administration of hHK-1(4-11) in mice. Representative amplification curves from the ABI7300 System Software for NK_1_ receptor mRNAs, PPT-A and PPT-C mRNAs, respectively (a, b and c). Histogram showing the relative mRNA expression levels of the NK_1_ receptor, PPT-A and PPT-C, respectively (A, B and C), normalized with the housekeeping gene GAPDH mRNA expression in three independent experiments. All reactions were run in triplicate and independently repeated at least three times. Each value is normalized to that of the control group injected with normal saline, which was set to a ratio of 1, and represents the mean ± SEM. There was no significant difference from the respective control.

In order to compare the mRNA levels of NK_1_ receptors and different preprotachykinins, real-time quantitative RT-PCR was performed (Figure 2). The mRNA levels of NK_1_ receptors and preprotachykinins were normalized to that of the housekeeping gene GAPDH. The 2^−ΔΔCt^ method was used as a convenient way to analyze the relative changes in gene expression from real-time quantitative PCR experiments. Compared with the control group (normal saline group, normalized to 1), the mRNA levels of NK_1_ receptors after i.c.v. injection of hHK-1(4-11) at 5, 10, and 20 min were 0.91±0.05, 0.93±0.04, and 0.87±0.06, respectively (Figure 2a, A). The mRNA levels of PPT-A at 5, 10, and 20 min were 0.99±0.06, 0.93±0.17, and 0.96±0.09, respectively (Figure 2b, B). The mRNA levels of PPT-C at 5, 10, and 20 min were 0.96±0.08, 1.10±0.11, and 0.96±0.03, respectively (Figure 2c, C). Statistical analysis showed no difference between the control group and the hHK-1(4-11)-treated group at all time points, indicating that the mRNA expression of NK_1_ receptors and preprotachykinins may not have been affected by the i.c.v. injection of hHK-1(4-11) in mice.

### Characterization of the mRNAs Encoding Endogenous Opioid Receptors and Opioid Peptides

**Figure 3:**
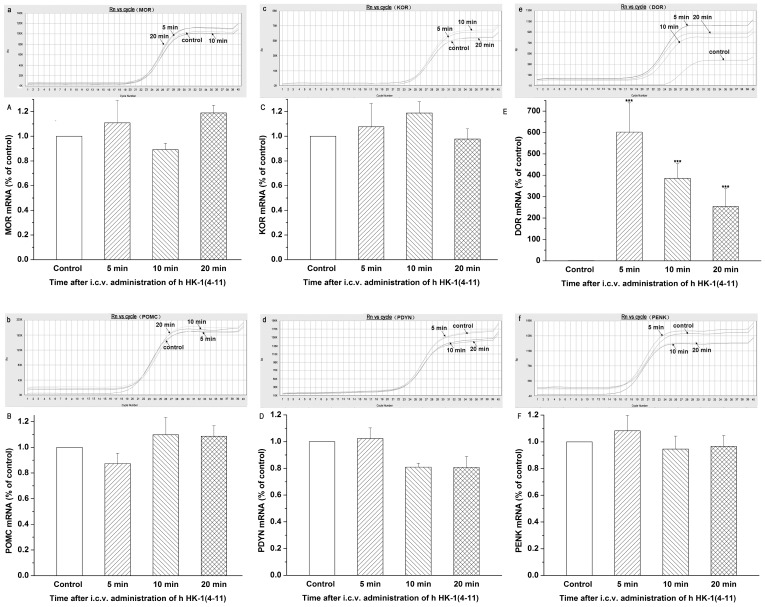
Quantitative determination of the mRNA expression levels of the endogenous opioid receptors and endogenous opioid peptides after i.c.v. administration of r/mHK-1 in mice. Representative amplification curves from the ABI7300 System Software for MOR (μ-opioid receptor), POMC (proopiomelanocortin), KOR (κ-opioid receptor),PDYN (prodynorphin), DOR (δ-opioid receptor) and PENK (proenkephalin) mRNAs, respectively (a, b, c, d, e and f). Histogram showing the relative mRNA expression levels of MOR, POMC, KOR, PDYN, DOR and PENK, respectively (A, B,C, D, E and F), normalized with the housekeeping gene GAPDH mRNA expression in three independent experiments. The bars correspond to the standard error of mean. Significantly different from the respective control: ***p≤0.001.

Using real-time quantitative RT-PCR technology, we found that the mRNA level of DOR in the hHK-1(4-11)-treated group was significantly enhanced in comparison with the control group. The values at 5, 10, and 20 min after i.c.v. administration of hHK-1(4-11) were 601.7±137.0, 386.1±83.1, and 254.3±68.7, respectively (Figure 3e, E). However, the mRNA levels of PENK (an endogenous ligand for DOR) did not significantly change since the values were 1.08±0.11, 0.94±0.10, and 0.97±0.08 at 5, 10 and 20 min after i.c.v. administration of hHK-1(4-11), respectively (Figure 3b, B). The mRNA levels of MOR (Figure 3a, A), POMC (an endogenous ligand for MOR, Figure 3b, B), KOR (Figure 3c, C) and PDYN (an endogenous ligand for KOR, Figure 3d, D), were also not significantly affected by i.c.v. administration of hHK-1(4-11) compared with i.c.v. administration of normal saline (control group).

### The Protein Expression of DOR Receptor was Significantly Increased by i.c.v. Administration of h HK-1(4-11) in Conscious Mice

**Figure 4:**
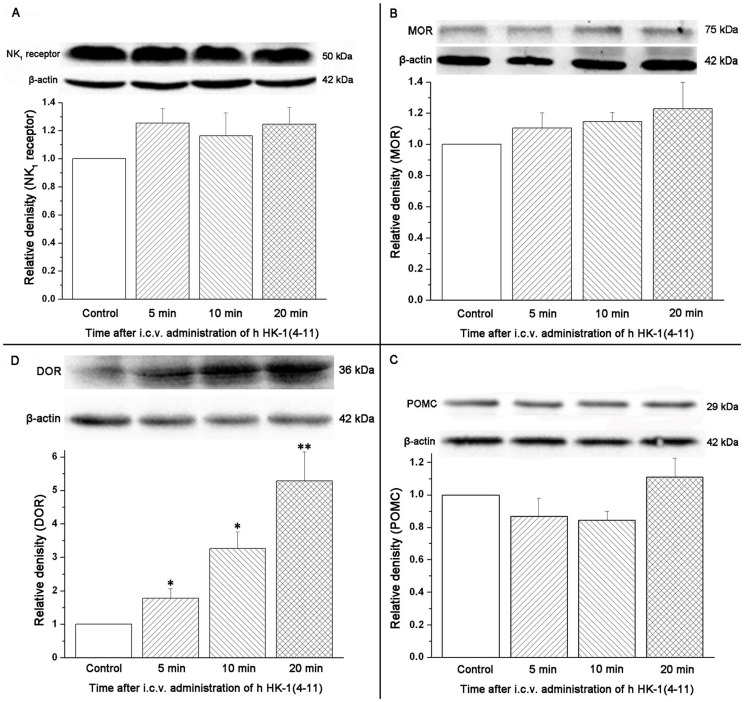
Semi-quantitative determination of the protein expression levels of NK_1_ receptor, MOR, POMC and DOR using the Western blotting technology after i.c.v. administration of hHK-1(4-11) compared with the control group in mice. The inset shows representative Western blots from at least three independent experiments. The bar graphs depict the relative protein expression levels of NK1 receptor (A), MOR (B), POMC (C) and DOR (D), respectively, normalized with the housekeeping protein β-actin expression in three independent experiments. The bars correspond to the standard error of mean. Significantly different from the respective control: *p≤0.05; **p≤0.01.

Finally, we sought to examine whether the up-regulation of DOR transcripts induced by i.c.v. administration of hHK-1(4-11) was associated with an elevation of DOR protein expression. As seen in Figure 4D, a significant increase in DOR protein expression was demonstrated in the hHK-1(4-11)-treated group compared with the control group. The protein expression levels of DOR at 5, 10, and 20 min were 1.78±0.28, 3.26±0.50, and 5.29±0.86, respectively (Figure 4D). Additionally, we examined whether the transcript expressions of NK_1_ receptor, MOR and POMC were associated with the expressions of their respective proteins. In Figure 4A–C, no significant change was detected between the hHK-1-(4-11)-treated group and the control group.

## Discussion

Similar to other small bioactive peptides, tachykinins are generated through proteolytic cleavage of larger precursors called preprotachykinins (PPTs) [26]. Unlike the mouse PPT-C gene in which the predicted HK-1 peptide is flanked by dibasic cleavage sites, the human PPT-C gene has two potential monobasic cleavage sites at the amino-terminal end of the predicted HK-1 peptide, and generate an 11 amino acid full-length peptide as well as an 8 amino acid truncated version (HK-1(4-11)) [4]. Severini et al. have summarized many published reports about the classical member of the tachykinin family, substance P (SP), and in this comprehensive review many results showed that the various peptide fragments of the SP molecule can exert opposite effects on a specific behavior [27]. For example, SP (1–7) inhibited not only nociception, but also aggressive and grooming behavior, while stimulating investigative motor behavior, like SP. The C-terminal peptide fragment [pGlu^6^] SP(7–11) exerted opposite effects to that of SP [27]. Considering the fact that peptide fragments of the SP molecule could exert different effects on a specific behavior, and that there is just one paper focusing on the effect of hHK-1(4-11) in the modulation of pain [19], it is essential and necessary to further investigate comparatively the effects of hHK-1(4-11) and its full length form on different behaviors in order to clarify the concrete effect and mechanism of PPT-C gene products *in vivo*. In this study, we further investigated the molecular mechanism and distribution sites of analgesic effects induced by i.c.v. administration of hHK-1(4-11), compared with the molecular mechanism and distribution sites of the peptide HK-1 [21].

Our present results showed that the expression levels of NK_1_ receptors and preprotachykinins were not affected by the i.c.v. injection of h HK-1(4-11), which is consistent with that of i.c.v. injection of r/m HK-1 [21]. Unlike r/m HK-1, the analgesic effect induced by i.c.v. administration of h HK-1(4-11) was not blocked by a unique series of opioid antagonists in vivo [19]. Correspondingly in our present work, the expression levels of MOR (μ opioid receptor) [28] and KOR (κ opioid receptor) [29], as well as their respective endogenous ligands POMC [30] and PDYN [30], were hardly changed after i.c.v. administration of h HK-1(4-11) compared with i.c.v. administration of normal saline (control group). Intriguingly, both the transcripts and proteins of DOR (δ opioid receptor) [31] were enhanced significantly in our present study, while the expression level of PENK (the endogenous ligand for DOR) [32] was not changed markedly. Taking our earlier results into account, in which the antagonist of DOR could markedly enhance the analgesic effect induced by i.c.v. administration of h HK-1(4-11) [19], our present results indicated that the counter-effect of δ receptors on the analgesia of h HK-1(4-11) may be partly relevant to the enhanced expression of δ opioid receptor. It remains an open question why i.c.v. administration of h HK-1(4-11) is able to elevate expression of DOR, as well as how this negative feedback is achieved. A variety of evidence suggests the involvement of periaqueductal central gray (PAG) neurons in the supraspinal effects of opioid-produced analgesia [33]–[37]. This indicates that the modulation of nociception by opiate analgesics may be mediated by a descending pathway involving the periaqueductal central gray. In our recent study, there was apparent and strong green fluorescence in the structure of PAG after i.c.v. administration of fluo-r/mHK-1 [21]. However, there was no apparent fluorescence in PAG structure after i.c.v. administration of fluo-hHK-1(4-11) in our present study. Considering the fact that there were discriminable mechanisms involved in the analgesic effects of i.c.v. administration of hHK-1(4-11) and r/mHK-1 mentioned above, our present results clearly indicated that the discriminable distribution sites in the central nervous system between hHK-1(4-11) and r/mHK-1 may be one major reason, especially the distribution site of PAG.

In summary, the major finding of the present study is the enhanced expression of δ opioid receptor after i.c.v. injection of hHK-1(4-11) in mouse model. It is interesting to consider our present results of hHK-1(4-11) with a comparison of r/mHK-1 in our published work, and the body of data derived from these experiments strongly suggest that the discriminable distribution sites in the central nervous system between hHK-1(4-11) and r/mHK-1 may be one major reason for the discriminable mechanisms of their respective analgesic effects. Of course, further experimental investigation is needed to uncover the mechanisms of why and how to engender these differences. In conclusion, our findings should facilitate the analysis of the complicated mechanisms involved in the nociceptive modulation of hemokinin-1 as well as its active fragments at the supraspinal level, and may lead to novel pharmacological interventions.
